# Waist-circumference-to-height-ratio had better longitudinal agreement with DEXA-measured fat mass than BMI in 7237 children

**DOI:** 10.1038/s41390-024-03112-8

**Published:** 2024-03-05

**Authors:** Andrew O. Agbaje

**Affiliations:** 1 Institute of Public Health and Clinical Nutrition, School of Medicine, Faculty of Health Sciences, University of Eastern Finland, Kuopio, Finland; 2 Children’s Health and Exercise Research Centre, Department of Public Health and Sports Sciences, Faculty of Health and Life Sciences, University of Exeter, Exeter, UK

## Abstract

**Background:**

The absolute agreement of surrogate measures of adiposity with dual-energy X-ray absorptiometry (DEXA)-measured body composition was examined.

**Methods:**

Over a 15-year follow-up, 7237 (3667 females) nine-year-old children from the Avon Longitudinal Study of Parents and Children (ALSPAC) UK birth cohort were included. Total fat mass (FM) and trunk FM were serially measured with DEXA at ages 9, 11, 15, 17, and 24 years. BMI and waist circumference-to-height ratio (WHtR) were computed. Pearson’s correlations, intraclass correlations (ICC), and area under curve (AUC) analyses were conducted.

**Results:**

Over 15 years, BMI, total FM, and trunk FM, increased but WHtR was relatively stable. WHtR provided a better longitudinal absolute agreement [males ICC 0.84 (95% CI 0.84–0.85); females 0.81 (0.80–0.82)] than BMI [(males (0.65 (0.64–0.66); females 0.72 (0.71–0.73)] with total FM as well as trunk FM from ages 9–24 years. WHtR cut-point for predicting excess total FM (75th–95th percentile) was 0.50–0.53 in males [AUC 0.86–0.94, sensitivity 0.51–0.79 and specificity 0.93–0.95]. WHtR cut-point for predicting excess total FM (75th–95th percentile) was 0.52–0.54 in females [AUC 0.83–0.95, sensitivity 0.38–0.68 and specificity 0.92–0.95]. Results were similar with trunk FM.

**Conclusion:**

WHtR is an inexpensive alternative to BMI for predicting FM in pediatrics.

**Impact:**

- Waist circumference-to-height ratio (WHtR) is a better adiposity surrogate measure than body mass index (BMI) in predicting fat mass and discriminating lean mass from childhood through young adulthood.
- BMI has been used as an inexpensive surrogate measure of adiposity in children for several decades. However, emerging findings suggest that BMI fails to discriminate between fat mass adiposity and lean mass.
- This is the first-ever longitudinal study in over 7000 children followed up for 15 years that identified WHtR as an inexpensive accurate measure that discriminates fat mass from lean mass that could replace BMI measure of obesity in pediatrics.

## Introduction

In 2016, over 340 million children and adolescents aged 5–19 years were overweight or obese necessitating urgent prevention of obesity and its associated consequences in the pediatric population.^1^ Excess fat or adiposity from childhood has been associated with insulin resistance, arterial stiffness, hypertension, and premature cardiovascular mortality.^2–8^ The classifications of overweight and obesity in the pediatric population have relied on anthropometrics and growth reference curves.^1,5^ The most universally accepted inexpensive and non-invasive assessment of adiposity is body mass index (BMI) for age, however, emerging studies with gold standard dual-energy Xray absorptiometry (DEXA) measure of adiposity suggest that increased BMI from childhood may not reflect fat mass but lean mass (muscle mass) and could overdiagnose obesity in children.^1,8–11^ The first ever American Academy of Pediatrics Clinical Practice Guideline for the evaluation and treatment of obesity in children published in January 2023 emphasized areas of knowledge gap such as research on alternative accurate measurements of adiposity in primary care.^12^ Other surrogate measures of adiposity employed in the pediatric population are waist circumference, waist circumference-to-height ratio (WHtR), height for age, weight for age, skin fold thickness, and BMI centiles.^13–17^

The correlations of surrogate measures of adiposity and DEXA measures of fat mass have largely been cross-sectional with few short-term longitudinal evidence.^13,17–22^ Moreover, none of these surrogate measures of adiposity have discriminated between objectively measured fat mass and lean mass.^13,18,20^ A cross-sectional study of 5355 children aged 8–19 years from the US National Health and Nutrition Surveys reported that WHtR performed better than BMI in detecting high body fat percentages.^21^ It remains controversial, however, whether WHtR better identifies excess fat mass adiposity than BMI in a longitudinal study of children and the appropriate cutoff.^13,14,17^ There is an increased call to revisit anthropometric indicators in the pediatric population for better surveillance of overweight and obesity.^23^ Longitudinal studies with objective and surrogate measures of body fat are important to account for and clarify the physiological and/or pathological changes that occur during growth and maturation from childhood through young adulthood in the same study population.^11^ Prospective studies also help to identify trends over a long period for which cross-sectional designs are limited.^11,21^ For example, Kakinami et al. in a 2-year follow-up study of 8–10-year-old children from Québec, Canada (*n* = 557) observed that changes in BMI were moderately correlated with changes in fat mass but waist circumference and WHtR were not measured.^18^ There are no existing long-term longitudinal studies on the relationships between surrogate and DEXA measures of adiposity from childhood through young adulthood which could enhance the identification of children at risk of cardiometabolic alterations during clinical and public health surveillance.^5,18,21,22,24,25^ This present study longitudinally examined the agreement of surrogate measures of adiposity with DEXA-measured fat mass and lean mass from childhood (age 9 years) through young adulthood (age 24 years) using data from the Avon Longitudinal Study of Parents and Children (ALSPAC) birth cohort, England, UK.

## Methods

### Study cohort

Details of the ALSPAC birth cohort have been published earlier.^6,26–28^ The ALSPAC birth cohort investigates factors that influence childhood development and growth. Altogether, 14,541 pregnancies from women residing in Avon, southwestern England, UK, who had a total of 14,676 foetuses, were enrolled between April 1, 1991, and December 31, 1992. When the oldest children were approximately 7 years of age, an attempt was made to bolster the initial sample with eligible cases who had failed to join the study originally resulting in 913 additional pregnancies. The total sample size for analyses using any data collected after 7 years of age was 15,447 pregnancies, resulting in 15,658 foetuses. Of these 14,901 were alive at 1 year of age. Regular clinic visits of the children commenced at 7 years of age and are still ongoing. Study data at 24 years were collected and managed using REDCap electronic data capture tools.^29^ For our analysis, we included 7237 participants who had complete height, weight, BMI, BMI standard deviation score (BMI-SDS), waist circumference, WHtR, total body fat mass, trunk fat mass, and total body lean mass at baseline age 9 years (Supplementary Fig. 1). Ethical approval for the study was obtained from the ALSPAC Ethics and Law Committee and the Local Research Ethics Committees. Informed consent for the use of data collected via questionnaires and clinics was obtained from participants following the recommendations of the ALSPAC Ethics and Law Committee at the time. Participants were invited to the clinic at their respective ages, 9, 11, 15, 17 and 24 years. Measurements were conducted at ages 9 years (baseline) and subsequent follow-up at age 11 years (2-year follow-up), age 15 years (4-year follow-up), age 17 years (8-year follow-up), and age 24 years clinic visits (15-year follow-up).

### Anthropometric and body composition

At 9, 11, 15, and 17 years clinic visits, participants’ height (meters) was measured using a stadiometer (SECA 213, Birmingham, UK) and weight (kilogram) using electronic weighing scales (Marsden M-110, Rotherham, UK).^6,7,9,30–32^ At 24 years clinic visit, standing height to the nearest meters was measured using a Harpenden wall-mounted stadiometer (Holtain Ltd, Crosswell, Crymych, UK), and weight to the nearest 0.1 kg at age 24 years was measured using Tanita TBF-401 (Model A, Tanita Corp., Tokyo, Japan) electronic body composition scales.^6,7,9^ BMI was computed as weight in kilograms per height in meters squared. BMI-SDS was derived using Lambda Mu Sigma & 1990 British Growth Reference at ages 9-, 11-, 15-, and 17-year clinic visits. Waist circumference was measured at ages 9-, 11-, 15-, and 24 years clinic visits to the nearest mm at the minimum circumference of the abdomen between the iliac crests and the lowest ribs, the tape was kept perpendicular to the long axis of the body, touching the skin but not compressing the tissue. WHtR for each clinic visit was computed as the ratio of waist circumference to height.

A Lunar Prodigy narrow fan beam densitometer was used to perform a whole body DEXA scan where bone content, lean, and fat masses are measured. Total body fat mass, trunk fat mass, and lean mass were measured using DEXA (Lunar Prodigy software version 15, GE Medical Systems, Madison, Wisconsin) at 9, 11, 15, 17, and 24-year clinic visits.^8,33–35^ The procedure was clearly explained to the participants and their consent was obtained before proceeding. The participants were asked to lie on the Prodigy couch (in light clothing without any metal fastenings), Height, weight, date of birth, sex, and ethnicity (if appropriate) were entered into the computer and the machine was started. The arm of the machine moved over the participants and two sources of X-ray scanned the participants. Once complete the tester examined the scan to ensure its quality and a picture of the skeleton part of the scan was printed out and given to the participant to keep (https://www.gehealthcare.com/products/bone-and-metabolic-health/body-composition). A daily quality assessment was performed using the calibration block in accordance with the manufacturer’s recommendations. The radiation protection supervisor or deputy scanned a spine phantom weekly. Each scan was manually screened for anomalies, motion, and material artefacts. Subregion edges and nodes were aligned manually according to specified criteria based on bony anatomical landmarks. Trunk fat mass was estimated using the automatic region of interest that included the chest, abdomen, and pelvis. The scans were visually inspected and realigned where necessary. Repeated DEXA measurements for 122 participants were performed on the same day, and the repeatability coefficient (twice the standard deviation of the difference between measurement occasions) for fat mass was 0.5 kg.^6,7,9,34,36^ At age 17–24 years, participants were excluded from the DEXA scan if they were pregnant, had a radiological investigation using contrast media within the week before the scan, had a recent nuclear medicine investigation with persistent radioactivity, and weight was greater than 159 kg. All participants attained puberty before age 17-year clinic visit, based on maturation assessed with time (years) to age at peak height velocity, an objective measure of pubertal or maturation status without having to rely on physical examination or self-report.^6,7,9^ This was derived using Superimposition by Translation And Rotation mixed-effects growth curve analysis.^6,7,9^

### Statistical analysis

Participant’s body composition characteristics were summarized as means and standard deviation. The normality of variables and sex differences were examined using independent *t* tests.

The cross-sectional and longitudinal correlation of surrogate measures of height, weight, BMI, BMI-SDS, waist circumference, and WHtR with total fat mass, trunk fat mass, and lean mass were examined. To assess the reliability and absolute agreement of different surrogate measures of body composition with DEXA measures, intraclass correlation coefficients were calculated in both cross-sectional and longitudinal analyses. Pediatric research and clinically useful sex-stratified total fat mass and trunk fat mass percentile cut points were determined at 5th, 10th,15th, 25th, 50th, 75th, 80th, 85th, 90th, and 95th percentiles, for each age at clinic visits and longitudinal cumulative values. Linear regressions of the longitudinal relationships between surrogate measures of body composition and DEXA measures based on sex were examined. The area under the curve from receiver-operating characteristic (ROC) analyses was used to longitudinally predict optimal cut points of WHtR to identify distinct excess total fat mass and trunk fat mass adiposity (75th, 85th, 90th, and 95th percentiles) during growth from childhood through young adulthood. The current obesity prevalence of 23% in children and adolescents in the UK was specified in the ROC model.^37^ The area under the curve with a 95% confidence interval, sensitivity, and specificity, as well as the 95% confidence intervals of the WHtR optimal cut points were presented. All ROC analyses were sex-based. The DeLong method was used in calculating the standard error of the area under the curve.^38^ Confidence interval was calculated using exact binomial confidence interval for the area under the curve and bootstrap confidence intervals with 1000 iterations were presented. As proposed by Swets, the classification of anthropometric indicators in relation to the discriminatory power by the area under the curve could be interpreted as follows; ≤0.5 is considered to have no discriminatory power, >0.5 and ≤0.7 has low discriminatory power, >0.7 and ≤0.9 has excellent discriminatory power, and 1 is a perfect test.^39^

Differences and associations with a two-sided *p* value < 0.05 were considered statistically significant with conclusions based on effect estimates and their confidence intervals. Analyses involving a sample of 5000 ALSPAC children at 0.8 statistical power, 0.05 alpha, and two-sided *p* value would show a minimum detectable effect size of 0.048 standard deviations if they had relevant exposure for a normally distributed quantitative variable.^40^ All statistical analyses were performed using SPSS Statistical Software, version 27.0 (IBM Corp, Armonk, NY) and MedCalc® Statistical Software, version 22.016 (MedCalc Software Ltd, Ostend, Belgium; https://www.medcalc.org; 2023).

## Results

### Cohort study characteristics

Altogether 14,901 children in the ALSPAC birth cohort were alive at 1 year of age, of whom 7722 children participated in the 9-year follow-up clinic visit, 7159 children participated in the 11-year follow-up clinic visit, 5509 adolescents participated in the 15-year follow-up clinic visit, 5217 adolescents participated in the 17-year follow-up clinic visit, and 4026 young adults participated in the 24-year follow-up clinic visit (Supplementary Fig. 1). Only 7237 participants who had complete surrogate adiposity and DEXA measurements at age 9 years were included in the study. All measures of body composition increased from ages 9 through 24 years, except WHtR which was stable from age 9–15 years and slightly increased at age 24 years (Table 1). WHtR measure was not available at the age 17-year clinic visit. Females had more total fat mass and trunk fat mass and less lean mass than males. Females had more weight at ages 9 and 11 years than males which became less than males at ages 15, 17, and 24 years (Table 1). Waist circumference was higher in males than females from 9 through 24 years. Other characteristics of our study participants are shown in Table 1.

**Table 1:** Descriptive characteristics of cohort participants.

Clinic visit	9 years (*N* = 7237)			11 years (*N* = 6004)			15 years (*N* = 3642)		
Variables	Male (*n* = 3570)	Female (*n* = 3667)	*P* value	Male (*n* = 2940)	Female (*n* = 3064)	*P* value	Male (*n* = 1706)	Female (*n* = 1936)	*P* value
	Mean (SD)	Mean (SD)		Mean (SD)	Mean (SD)		Mean (SD)	Mean (SD)	
Age at clinic visit (years)	9.88 (0.33)	9.88 (0.32)	0.970	11.72 (0.22)	11.74 (0.23)	0.077	15.40 (0.24)	15.43 (0.28)	<0.001
Height (m)	1.40 (0.06)	1.39 (0.07)	0.001	1.50 (0.07)	1.51 (0.07)	<0.001	1.74 (0.08)	1.65 (0.06)	<0.0001
Weight (kg)	34.44 (7.18)	35.07 (7.75)	<0.001	42.69 (0.10)	44.57 (10.24)	<0.001	63.60 (11.59)	58.31 (9.90)	<0.001
Body mass index (kg/m^2^)	17.5 (2.78)	17.93 (2.99)	<0.001	18.81 (3.30)	19.31 (3.49)	<0.001	20.84 (3.12)	21.45 (3.26)	<0.001
Body mass index-SDS	0.33 (1.13)	0.27 (1.13)	0.018	0.41 (1.19)	0.30 (1.19)	<0.001	0.31 (1.04)	0.30 (1.03)	0.460
Waist circumference (cm)	63.23 (7.74)	62.63 (7.93)	0.001	68.69 (9.76)	67.98 (9.22)	0.004	76.90 (8.91)	76.39 (8.85)	0.077
Waist-to-height ratio	0.45 (0.05)	0.45 (0.05)	0.017	0.46 (0.06)	0.45 (0.06)	<0.001	0.44 (0.05)	0.46 (0.05)	<0.001
Total fat mass (kg)	7.39 (4.91)	9.74 (5.14)	<0.001	10.42 (6.67)	13.0 (6.77)	<0.001	10.95 (7.81)	18.35 (0.75)	<0.001
Trunk fat mass (kg)	2.90 (2.33)	4.02 (2.57)	<0.001	4.30 (3.24)	5.65 (3.44)	<0.001	5.06 (4.13)	8.55 (4.11)	<0.001
Lean mass (kg)	25.52 (3.0)	23.67 (3.20)	<0.001	30.19 (4.21)	29.30 (4.48)	<0.001	49.73 (6.73)	36.94 (3.92)	<0.0001

Clinic visit	17 years (*N* = 3981)			24 years (*N* = 3035)		
Variables	Male (*n* = 1786)	Female (*n* = 2195)	P-value	Male (*n* = 1189)	Female (*n* = 1846)	*P* value
	Mean (SD)	Mean (SD)		Mean (SD)	Mean (SD)	
Age at clinic visit (years)	17.76 (0.38)	17.77 (0.39)	0.104	24.52 (0.78)	24.42 (0.79)	0.001
Height (m)	1.79 (0.07)	1.65 (0.63)	<0.0001	1.80 (0.67)	1.66 (0.06)	<0.0001
Weight (kg)	72.27 (13.38)	62.56 (12.74)	<0.001	80.42 (14.93)	68.23 (14.88)	<0.001
Body mass index (kg/m^2^)	22.55 (3.84)	22.89 (4.38)	0.012	24.77 (4.30)	24.74 (5.14)	0.909
Body mass index-SDS	0.37 (1.17)	0.37 (1.23)	0.966	NA		
Waist circumference (cm)	NA			86.06 (11.64)	78.17 (12.01)	<0.001
Waist-to-height ratio	NA			0.48 (0.06)	0.47 (0.07)	0.005
Total fat mass (kg)	13.89 (9.95)	21.43 (9.49)	<0.001	20.58 (9.81)	24.76 (10.70)	<0.001
Trunk fat mass (kg)	7.36 (5.66)	10.78 (5.41)	<0.001	10.51 (5.99)	11.51 (6.07)	<0.001
Lean mass (kg)	55.15 (6.33)	37.99 (4.22)	<0.0001	56.92 (7.44)	41.19 (5.32)	<0.0001

The values are means (standard deviations). Differences between sexes were tested using Student’s *t* test. *P* value for sex differences <0.05 is considered statistically significant.

*SDS* Standard Deviation score for BMI using LMS & 1990 British Growth Reference, *NA* not available.

### Longitudinal associations of surrogate body composition measures with DEXA measures

All longitudinal measures of body composition were positively associated with total fat mass, trunk fat mass, and lean mass except BMI-SDS which was inversely associated in both males and females (Table 2). BMI had the largest standardized coefficients in the positive associations with total fat mass and trunk fat mass in both males and females while height and weight had the least associations in males and females, respectively. Height had the largest standardized coefficients in the positive associations with lean mass while WHtR had the least associations with lean mass in both males and females (Table 2). Accounting for puberty or somatic maturation as objectively expressed by time (years) to age at peak height velocity did not significantly alter the results (data not shown).

**Table 2:** Longitudinal associations between surrogate measures of body composition and DEXA-measured fat mass and lean mass cumulatively measured from ages 9–24 years.

Variables	Male				Female			
	*B* (95% CI)	*β* (standardized)	*p* value	*R*^2^	B (95% CI)	*β* (standardized)	*p* value	*R*^2^
Total fat mass (kg)								
Height (m)	0.035 (0.034–0.036)	0.425	<0.0001	0.181	0.037 (0.036–0.038)	0.578	<0.0001	0.334
Weight (kg)	3.021 (2.974–3.067)	0.763	<0.0001	0.582	2.901 (2.882–2.921)	0.929	<0.0001	0.864
Body mass index (kg/m^2^)	1.541 (1.528–1.554)	0.909	<0.0001	0.827	1.359 (1.351–1.366)	0.947	<0.0001	0.898
Body mass index-SDS	−0.553 (−0.661–−0.446)	−0.109	<0.001	0.012	−0.520 (−0.602–−0.438)	−0.124	<0.001	0.015
Waist circumference (cm)	1.754 (1.697–1.812)	0.551	<0.0001	0.303	1.175 (1.129–1.221)	0.454	<0.0001	0.206
Waist-to-height ratio	0.004 (0.004–0.005)	0.730	<0.0001	0.532	0.004 (0.004–0.004)	0.678	<0.0001	0.460
Trunk fat mass (kg)								
Height (m)	0.069 (0.067–0.071)	0.464	<0.0001	0.215	0.066 (0.064–0.067)	0.559	<0.0001	0.312
Weight (kg)	5.674 (5.595–5.752)	0.795	<0.0001	0.632	5.259 (5.220–5.299)	0.914	<0.0001	0.836
Body mass index (kg/m^2^)	2.792 (2.770–2.815)	0.914	<0.0001	0.836	2.470 (2.454–2.486)	0.935	<0.0001	0.874
Body mass index-SDS	−1.139 (−1.328–−0.949)	−0.126	<0.001	0.016	−0.954 (−1.104–−0.803)	−0.124	<0.001	0.015
Waist circumference (cm)	3.026 (2.924–3.129)	0.536	<0.0001	0.287	2.066 (1.981–2.152)	0.437	<0.0001	0.191
Waist-to-height ratio	0.008 (0.008–0.008)	0.709	<0.0001	0.502	0.007 (0.007–0.007)	0.694	<0.0001	0.481
Lean mass (kg)								
Height (m)	0.047 (0.047–0.048)	0.951	<0.0001	0.905	0.071 (0.071–0.072)	0.908	<0.0001	0.825
Weight (kg)	2.197 (2.179–2.214)	0.919	<0.0001	0.844	3.429 (3.399–3.459)	0.893	<0.0001	0.798
Body mass index (kg/m^2^)	0.678 (0.665–0.692)	0.663	<0.0001	0.440	1.208 (1.186–1.230)	0.685	<0.0001	0.469
Body mass index-SDS	−0.643 (−0.707–−0.580)	−0.210	<0.001	0.044	−0.886 (−0.984–−0.787)	−0.175	<0.001	0.030
Waist circumference (cm)	0.626 (0.587–0.665)	0.326	<0.001	0.107	1.005 (0.946–1.064)	0.321	<0.001	0.103
Waist-to-height ratio	0.001 (<0.0001–0.001)	0.139	<0.001	0.019	0.002 (0.002–0.002)	0.245	<0.001	0.060

A 2-sided *p* value < 0.005 was considered statistically significant.

*B* unstandardized regression coefficients from linear regression analysis, *CI* confidence value, *SDS* standard deviation score for BMI using LMS & 1990 British Growth Reference.

### Longitudinal correlations and absolute agreements of body composition measures with DEXA measures

BMI had the highest correlation with total fat mass and trunk fat mass in both males and females while height and weight had the least associations in males and females, respectively (Table 3). Height had the largest standardized coefficients in the positive associations with lean mass while WHtR had the least associations with lean mass in both males and females (Table 3). BMI-SDS was negatively correlated with all DEXA measures. WHtR and weight had the highest absolute agreement (0.81–0.89) with total fat mass and trunk fat mass, but WHtR had the least absolute agreement with lean mass (Table 3). BMI had a moderate absolute agreement with total fat mass and trunk fat mass (0.65–0.72). The cross-sectional correlation and absolute agreement analyses for each age at clinic visits were similar to the longitudinal analyses (Tables 4 and 5).

**Table 3:** Longitudinal Pearson correlation coefficient and intraclass correlation absolute agreement between surrogate measures of body composition and DEXA-measured fat mass and lean mass cumulatively measured from ages 9–24 years.

	Total fat mass				Trunk fat mass				Lean mass			
Variables	Male		Female		Male		Female		Male		Female	
	*r* (*p* value)	ICC (CI)	*r* (*p* value)	*ICC (CI)*	*r* (*p* value)	ICC (CI)	*r* (*p* value)	ICC (CI)	*r* (*p* value)	ICC (CI)	*r* (*p* value)	ICC (CI)
Height	0.43 (<0.0001)	0.34 (0.32–0.36)	0.58 (<0.001)	0.39 (0.36–0.40)	0.46 (<0.0001)	0.37 (0.35–0.39)	0.56 (<0.001)	0.37 (0.35–0.39)	0.95 (<0.0001)	0.63 (0.62–0.65)	0.91 (<0.0001)	0.54 (0.52–0.55)
Weight	0.76 (<0.0001)	0.81 (0.80–0.81)	0.93 (<0.0001)	0.89 (0.88–0.89)	0.80 (<0.0001)	0.83 (0.82–0.83)	0.91 (<0.0001)	0.87 (0.87–0.80)	0.92 (<0.0001)	0.90 (0.89–0.90)	0.89 (<0.0001)	0.86 (0.86–0.87)
Body mass index	0.91 (<0.0001)	0.65 (0.64–0.66)	0.95 (<0.0001)	0.72 (0.71–0.73)	0.91 (<0.0001)	0.66 (0.64–0.67)	0.94 (<0.0001)	0.71 (0.70–0.72)	0.66 (<0.001)	0.52 (0.50–0.54)	0.69 (<0.0001)	0.58 (0.56–0.59)
Body mass index-SDS	−0.11 (<0.0001)	−0.17 (−0.25–−0.09)	−0.12 (<0.0001)	−0.23 (−0.30–−0.17)	−0.13 (<0.001)	−0.20 (−0.29–−0.11)	−0.12 (<0.001)	−0.23 (−0.30–−0.17)	−0.21 (<0.001)	−0.35 (−0.51–−0.21)	−0.18 (<0.001)	−0.34 (−0.43–−0.25)
Waist circumference	0.55 (<0.0001)	0.64 (0.56–0.70)	0.45 (<0.0001)	0.58 (0.54–0.62)	0.54 (<0.0001)	0.63 (0.55–0.68)	0.44 (<0.0001)	0.57 (0.52–0.61)	0.33 (<0.0001)	0.43 (0.36–0.49)	0.32 (<0.001)	0.45 (0.41–0.48)
Waist-to-height ratio	0.73 (<0.0001)	0.84 (0.84–0.85)	0.68 (<0.0001)	0.81 (0.80–0.82)	0.71 (<0.0001)	0.83 (0.82–0.84)	0.69 (<0.0001)	0.82 (0.81–0.83)	0.14 (<0.001)	0.24 (0.21–0.28)	0.25 (<0.001)	0.39 (0.37–0.42)

*DEXA* dual-energy Xray absorptiometry, *SDS* standard deviation score.

**Table 4:** Cross-sectional Pearson’s correlation between surrogate measures of body composition and DEXA measured fat mass and lean mass for each clinic visit.

	Total fat mass (kg)		Trunk fat mass (kg)		Lean mass (kg)	
Variables	Male	Female	Male	Female	Male	Female
	*r* (*p* value)	*r* (*p* value)	*r* (*p* value)	*r* (*p* value)	*r* (*p* value)	*r* (*p* value)
9 years (*N* = 7237)						
Height (m)	0.44 (<0.001)	0.46 (<0.001)	0.41 (<0.001)	0.43 (<0.001)	0.82 (<0.0001)	0.83 (<0.0001)
Weight (kg)	0.93 (<0.0001)	0.94 (<0.0001)	0.91 (<0.0001)	0.92 (<0.0001)	0.78 (<0.0001)	0.83 (<0.0001)
Body mass index (kg/m^2^)	0.94 (<0.0001)	0.95 (<0.0001)	0.93 (<0.0001)	0.94 (<0.0001)	0.57 (<0.001)	0.61 (<0.0001)
Body mass index-SDS	0.87 (<0.0001)	0.90 (<0.0001)	0.85 (<0.0001)	0.89 (<0.0001)	0.58 (<0.0001)	0.59 (<0.0001)
Waist circumference (m)	0.93 (<0.0001)	0.92 (<0.0001)	0.93 (<0.0001)	0.92 (<0.0001)	0.59 (<0.0001)	0.64 (<0.0001)
Waist-to-height ratio	0.86 (<0.0001)	0.84 (<0.0001)	0.88 (<0.0001)	0.85 (<0.0001)	0.33 (<0.001)	0.37 (<0.0001)
11 years (*N* = 6004)						
Height (m)	0.40 (<0.001)	0.38 (<0.001)	0.37 (<0.001)	0.36 (<0.001)	0.83 (<0.0001)	0.83 (<0.0001)
Weight (kg)	0.92 (<0.0001)	0.92 (<0.0001)	0.90 (<0.0001)	0.91 (<0.0001)	0.76 (<0.0001)	0.80 (<0.0001)
Body mass index (kg/m^2^)	0.95 (<0.0001)	0.95 (<0.0001)	0.94 (<0.0001)	0.94 (<0.0001)	0.53 (<0.001)	0.58 (<0.0001)
Body mass index-SDS	0.88 (<0.0001)	0.90 (<0.0001)	0.87 (<0.0001)	0.89 (<0.0001)	0.54 (<0.0001)	0.58 (<0.0001)
Waist circumference (m)	0.94 (<0.0001)	0.92 (<0.0001)	0.94 (<0.0001)	0.93 (<0.0001)	0.54 (<0.0001)	0.57 (<0.0001)
Waist-to-height ratio	0.88 (<0.0001)	0.85 (<0.0001)	0.90 (<0.0001)	0.86 (<0.0001)	0.29 (<0.001)	0.29 (<0.0001)
15 years (*N* = 3642)						
Height (m)	0.16 (<0.001)	0.21 (<0.001)	0.18 (<0.001)	0.19 (<0.001)	0.76 (<0.0001)	0.64 (<0.001)
Weight (kg)	0.80 (<0.0001)	0.92 (<0.0001)	0.82 (<0.0001)	0.90 (<0.0001)	0.70 (<0.001)	0.65 (<0.001)
Body mass index (kg/m^2^)	0.88 (<0.0001)	0.92 (<0.0001)	0.88 (<0.0001)	0.90 (<0.0001)	0.43 (<0.001)	0.42 (<0.001)
Body mass index-SDS	0.79 (<0.0001)	0.88 (<0.0001)	0.79 (<0.0001)	0.86 (<0.0001)	0.50 (<0.001)	0.42 (<0.001)
Waist circumference (m)	0.89 (<<0.0001)	0.93 (<0.0001)	0.90 (<0.0001)	0.83 (<0.0001)	0.40 (<0.001)	0.41 (<0.001)
Waist-to-height ratio	0.86 (<0.0001)	0.78 (<0.0001)	0.87 (<0.0001)	0.78 (<0.0001)	0.11 (<0.001)	0.21 (<0.001)
17 years (*N* = 3981)						
Height (m)	0.10 (<0.001)	0.16 (<0.001)	0.09 (<0.001)	0.49 (<0.001)	0.61 (<0.001)	0.61 (<0.001)
Weight (kg)	0.88 (<0.0001)	0.91 (<0.0001)	0.88 (<0.0001)	0.90 (<0.0001)	0.61 (<0.001)	0.63 (<0.001)
Body mass index (kg/m^2^)	0.91 (<0.0001)	0.90 (<0.0001)	0.91 (<0.0001)	0.89 (<0.0001)	0.40 (<0.001)	0.43 (<0.001)
Body mass index-SDS	0.82 (<0.0001)	0.90 (<0.0001)	0.82 (<0.0001)	0.84 (<0.0001)	0.45 (<0.001)	0.42 (<0.001)
24 years (*N* = 3035)						
Height (m)	0.11 (<0.001)	0.12 (<0.001)	0.08 (0.010)	0.09 (<0.001)	0.53 (<0.001)	0.55 (<0.001)
Weight (kg)	0.88 (<0.0001)	0.94 (<0.0001)	0.87 (<0.0001)	0.92 (<0.0001)	0.79 (<0.001)	0.77 (<0.001)
Body mass index (kg/m^2^)	0.89 (<0.0001)	0.93 (<0.0001)	0.89 (<0.0001)	0.93 (<0.0001)	0.61 (<0.001)	0.60 (<0.001)
Waist circumference (m)	0.92 (<0.0001)	0.90 (<0.0001)	0.93 (<0.0001)	0.92 (<0.0001)	0.54 (<0.001)	0.60 (<0.001)
Waist-to-height ratio	0.88 (<0.0001)	0.87 (<0.0001)	0.90 (<0.0001)	0.88 (<0.0001)	0.39 (<0.001)	0.47 (<0.001)

*r* Pearsons correlation coefficient.

**Table 5:** Absolute agreement of intraclass cross-sectional correlation of waist circumference-to-height ratio and body mass index and DEXA measured fat mass and lean mass for each clinic visit.

	Total fat mass (kg)		Trunk fat mass (kg)		Lean mass (kg)	
*Variables*	Male	Female	Male	Female	Male	Female
	ICC (95% CI)	ICC (95% CI)	ICC (95% CI)	ICC (95% CI)	ICC (95% CI)	ICC (95% CI)
9 years (*N* = 7237)						
Body mass index (kg/m^2^)	0.970 (0.968–0.972)	0.974 (0.972–0.76)	0.966 (0.964–0.968)	0.969 (0.967–0.971)	0.723 (0.704–0.741)	0.755 (0.739–0.770)
Waist-to-height ratio	0.926 (0.921–0.931)	0.911 (0.905–0.916)	0.935 (0.930–0.939)	0.920 (0.915–0.925)	0.499 (0.465–0.531)	0.543 (0.512–0.571)
11 years (*N* = 6004)						
Body mass index (kg/m^2^)	0.972 (0.970–0.974)	0.974 (0.972–0.976)	0.970 (0.967–0.972)	0.970 (0.967–0972)	0.690 (0.666–0.711)	0.732 (0.712–0.750)
Waist-to-height ratio	0.937 (0.933–0.942)	0.917 (0.911–0.923)	0.946 (0.942–0.949)	0.926 (0.921–0.931)	0.449 (0.407–0.487)	0.453 (0.412–0.490)
15 years (*N* = 3642)						
Body mass index (kg/m^2^)	0.942 (0.937–0.947)	0.965 (0.962–0.968)	0.946 (0.941–0.950)	0.958 (0.954–0.961)	0.590 (0.554–0.623)	0.612 (0.580–0.643)
Waist-to-height ratio	0.925 (0.918–0.932)	0.870 (0.857–0.881)	0.927 (0.920–0.934)	0.875 (0.863–0.887)	0.203 (0.123–0.275)	0.347 (0.287–0.403)
24 years (*N* = 3035)						
Body mass index (kg/m^2^)	0.943 (0.936–0.949)	0.966 (0.962–0.969)	0.943 (0.936–0.949)	0.961 (0.957–0.964)	0.760 (0.731–0.786)	0.753 (0.729–0.774)
Waist-to-height ratio	0.937 (0.930–0.944)	0.928 (0.921–0.934)	0.946 (0.940–0.952)	0.941 (0.935–0.946)	0.564 (0.511–0.611)	0.637 (0.602–0.668)

### Receiver operating curve analyses for predicting excess total fat mass adiposity from WHtR

**Fig. 1:**
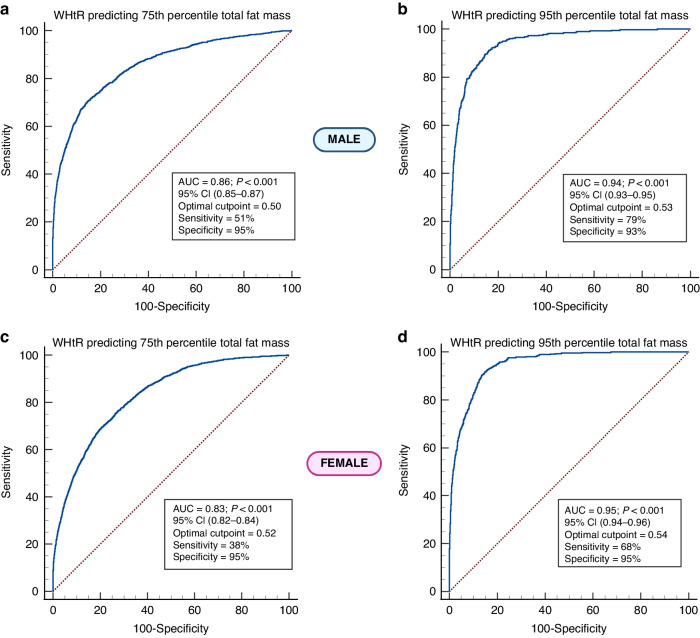
Predicting excess total body fat mass with waist circumference-to-height ratio. Receiver operating characteristics curve analyses of waist circumference-to-height ratio optimal cut-point for longitudinally predicting excess adiposity at the 75th and 95th percentile of total fat mass in males (**a**, **b**) and females (**c**, **d**), respectively. AUC area under the curve, CI confidence interval, WHtR waist circumference-to-height ratio. To derive an optimal cutpoint, the prevalence of obesity was set at 23% based on the latest evidence in the UK pediatric population.

The area under the curve of 0.86 identified 0.50 in males and 0.52 in females as WHtR cutpoints that longitudinally predicted the 75th percentile of total fat mass with a sensitivity of 0.51 in males and 0.38 in females and a specificity of 0.95 in both males and females (Table 6 and Fig. 1). The area under the curve of 0.94 identified 0.53 in males and 0.54 in females as WHtR optimal cutpoints that longitudinally predicted the 95th percentile of total fat mass with a sensitivity of 0.79 in males and 0.68 in females and a specificity of 0.93 in males and 0.95 in females (Table 6 and Fig. 1). The WHtR optimal cutpoints and area under the curve steadily increased with increasing percentile of total fat mass or trunk fat mass. The sex-based total fat mass results were similar to trunk fat mass results (Table 6).

**Table 6:** Receiver operating curve analysis for longitudinally predicting excess total fat mass or trunk fat mass with waist circumference-to-height ratio optimal cutpoint from ages 9 through 24 years.

Sex	AUC (95% CI)	Sensitivity	Specificity	WHtR optimal cutpoint (95% CI)
75th percentile for excess total body fat				
Male	0.856 (0.845–0.866)	0.512	0.946	0.500 (0.488–0.513)
Female	0.830 (0.820–0.838)	0.384	0.948	0.516 (0.501–0.525)
85th percentile for excess total body fat				
Male	0.885 (0.875–0.896)	0.554	0.937	0.512 (0.497–0.529)
Female	0.878 (0.868–0.887)	0.537	0.932	0.515 (0.506–0.523)
90th percentile for excess total body fat				
Male	0.902 (0.890–0.912)	0.577	0.944	0.525 (0.514–0.530)
Female	0.910 (0.900–0.918)	0.651	0.924	0.517 (0.505–0.524)
95th percentile for excess total body fat				
Male	0.942 (0.930–0.951)	0.794	0.929	0.525 (0.524–0.529)
Female	0.948 (0.940–0.955)	0.675	0.953	0.544 (0.517–0.552)
75th percentile for excess trunk body fat				
Male	0.837 (0.826–0.848)	0.421	0.961	0.512 (0.504–0.525)
Female	0.838 (0.829–0.847)	0.502	0.919	0.500 (0.497–0.515)
85th percentile for excess trunk body fat				
Male	0.879 (0.868–0.889)	0.541	0.933	0.511 (0.490–0.525)
Female	0.880 (0.870–0.889)	0.539	0.932	0.515 (0.509–0.534)
90th percentile for excess trunk body fat				
Male	0.894 (0.881–0.904)	0.627	0.919	0.512 (0.496–0.523)
Female	0.918 (0.909–0.925)	0.681	0.922	0.515 (0.504–0.524)
95th percentile for excess trunk body fat				
Male	0.942 (0.931–0.950)	0.782	0.929	0.525 (0.513–0.529)
Female	0.951 (0.942–0.958)	0.759	0.941	0.536 (0.520–0.544)

To derive optimal cutpoint, the prevalence of obesity was set at 23% based on the latest evidence in the UK pediatric population.

*AUC* area under the curve, *CI* confidence interval, *WHtR* waist circumference-to-height ratio.

### Percentiles references for WHtR, total fat mass, and trunk fat mass

**Fig. 2:**
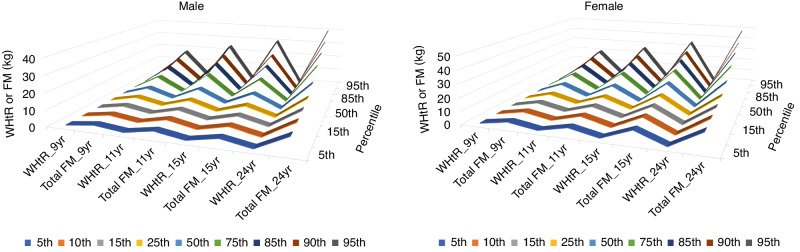
The distribution of waist circumference-to-height ratio relative to total body fat mass. Mean distribution of waist circumference-to-height ratio (WHtR) and total fat mass (FM) during growth from childhood (age 9 years) through young adulthood (age 24 years) in males and females across different percentiles. Females had significantly higher total fat mass than males as evidenced by the less flat contour ridges in childhood.

The longitudinal 75th percentile for WHtR in males and females is ~0.48, while the total fat mass 75th percentile in males is 14.91 kg and 21.95 kg in females (Table 7 and Fig. 2). The longitudinal 75th percentile for trunk fat mass in males and females is 7.05 kg and 10.44 kg in females (Table 7 and Fig. 2).

**Table 7:** Percentile cut-points for waist circumference-to-height ratio and total fat mass and trunk fat mass among males and females at each clinic visit and cumulative values.

	Males									Females								
*Percentile*	5th	10th	15th	25th	50th	75th	85th	90th	95th	5th	10th	15th	25th	50th	75th	85th	90th	95th
Waist circumference to height ratio										Waist circumference to height ratio								
Age 9 years	0.397	0.405	0.410	0.420	0.440	0.470	0.497	0.519	0.553	0.386	0.396	0.403	0.413	0.440	0.475	0.500	0.519	0.550
Age 11 years	0.392	0.401	0.407	0.417	0.440	0.483	0.518	0.542	0.580	0.381	0.391	0.398	0.409	0.435	0.480	0.511	0.530	0.561
Age 15 years	0.388	0.395	0.401	0.409	0.429	0.456	0.487	0.506	0.547	0.393	0.404	0.413	0.426	0.455	0.495	0.518	0.537	0.564
Age 17 years	NA									NA								
Age 24 years	0.399	0.411	0.419	0.434	0.464	0.506	0.535	0.564	0.609	0.384	0.397	0.405	0.420	0.453	0.506	0.545	0.572	0.620
Age 9–24 y	0.393	0.402	0.408	0.418	0.440	0.477	0.508	0.531	0.568	0.385	0.396	0.403	0.415	0.443	0.485	0.514	0.536	0.566
Total fat mass (kg)										Total fat mass (kg)								
Age 9 years	2.414	2.935	3.300	3.977	5.751	9.314	12.124	14.426	17.413	3.737	4.420	4.954	5.954	8.543	12.228	14.798	16.897	19.963
Age 11 years	3.324	4.107	4.631	5.527	8.357	13.599	17.365	19.867	24.095	5.082	6.072	6.752	7.971	11.284	16.433	20.100	22.249	26.292
Age 15 years	3.752	4.302	4.800	5.768	8.418	13.553	18.646	22.839	30.134	8.913	10.507	11.480	13.317	17.282	22.495	26.445	29.328	34.495
Age 17 years	4.252	5.047	5.710	6.935	10.776	17.768	22.948	27.767	35.348	10.506	12.150	13.188	14.878	19.349	25.331	29.700	34.119	40.676
Age 24 years	9.407	10.858	11.980	13.768	18.173	24.924	29.789	34.059	39.620	13.135	14.457	15.482	17.320	21.976	29.193	35.221	39.981	47.587
Age 9–24 y	3.032	3.679	4.231	5.253	8.633	14.911	19.539	23.155	30.053	4.822	6.127	7.423	9.909	15.519	21.950	26.144	29.716	36.740
Trunk fat mass (kg)										Trunk fat mass (kg)								
Age 9 years	0.800	0.949	1.101	1.360	2.061	3.638	4.972	6.143	7.958	1.229	1.531	1.750	2.151	3.342	5.113	6.516	7.575	9.337
Age 11 years	1.131	1.431	1.619	2.001	3.159	5.647	7.478	9.030	11.279	1.911	2.256	2.547	3.103	4.644	7.277	9.186	10.387	12.837
Age 15 years	1.492	1.750	1.995	2.400	3.687	6.275	8.742	11.150	15.303	3.635	4.396	4.878	5.746	7.754	10.877	12.978	14.680	17.638
Age 17 years	2.061	2.442	2.783	3.462	5.618	9.311	12.394	14.886	19.548	4.788	5.553	6.151	7.102	9.492	12.765	15.658	17.909	22.083
Age 24 years	3.793	4.618	5.291	6.340	8.893	13.239	16.045	18.447	22.502	4.931	5.737	6.341	7.320	9.924	14.004	17.481	19.892	24.409
Age 9–24 y	0.999	1.309	1.551	2.028	3.725	7.047	9.731	11.859	15.922	1.680	2.239	2.844	4.005	6.937	10.441	12.784	14.878	18.838
Lean mass (kg)										Lean mass (kg)								
Age 9 years	20.882	21.827	22.536	23.486	25.372	27.439	28.511	29.392	30.716	19.093	19.927	20.557	21.491	23.322	25.471	26.882	27.850	29.629
Age 11 years	24.146	25.341	26.063	27.318	29.703	32.533	34.376	35.721	37.654	22.623	23.731	24.606	26.058	28.916	32.220	34.066	35.399	37.190
Age 15 years	37.930	41.231	42.935	45.528	49.868	54.140	56.318	58.237	60.563	30.729	32.137	33.061	34.360	36.779	39.478	41.084	42.229	43.737
Age 17 years	45.231	47.264	48.834	50.866	54.982	59.167	61.572	63.220	65.982	31.738	32.925	33.790	35.163	37.683	40.443	42.175	43.458	45.038
Age 24 years	44.979	47.689	49.323	51.937	56.402	61.862	64.339	66.529	69.388	33.585	34.897	36.064	37.578	40.650	44.308	46.359	47.886	50.599
Age 9–24 y	22.545	23.826	24.812	26.563	32.920	51.995	55.993	58.547	62.147	20.752	22.046	23.152	25.286	32.645	38.141	40.485	42.167	44.549

NA, not available

## Discussion

This study presents the first and largest longitudinal correlation and absolute agreement between surrogate measures of body composition and gold standard measures of total fat mass, trunk fat mass, and lean mass from childhood through young adulthood. While the most universally accepted inexpensive and non-invasive assessment of adiposity is BMI, it has a lesser absolute agreement with total fat mass and trunk fat mass compared to WHtR. Importantly, WHtR had the least correlation, association, and absolute agreement with DEXA-measured lean mass suggesting that WHtR may be an inexpensive and non-invasive assessment specific for fat mass adiposity. BMI measures of body composition did not significantly distinguish between fat mass and lean mass. This result fills the knowledge gap on alternative measures of adiposity recently identified by the American Academy of Pediatrics in the first-ever clinical practice guideline for the evaluation and treatment of obesity in children.^12^

### Comparison with previous studies

A systematic review of 27 cross-sectional studies published between 2004 and 2014 including 7–10-year-old children reported that BMI and waist circumference had a moderate correlation with percent body fat assessed with DEXA.^20^ However, a 2-year longitudinal study of 557 Canadian children with an average age of 9.6 years and with at least one obese biological parent concluded that change in BMI was highly correlated with change in body fat mass assessed with DEXA.^18^ The systematic review of 27 cross-sectional studies also found that WtHR was moderately correlated with percent body fat estimated using skinfolds and air displacement plethysmography.^20^ Another 2016 systematic review and meta-analysis of 5 studies published between 2013 and 2015 reported that BMI had a higher correlation with DEXA measures of total fat mass than WHtR which was confirmed in the present study.^13^ A systematic review and meta-analysis published in 2017 included 5 cross-sectional studies of children and adolescents from Japan, New Zealand, and Portugal, and concluded that both BMI and WHtR had high discriminatory power to identify high DEXA-measured body fat but only the study from Japan presented a WHtR cut-off for detecting high fat mass.^17^ However, the authors highlighted a significant limitation of the studies which is the unavailability of agreement analysis of these surrogate measures of adiposity with DEXA measures as well as cut-offs.^13,17^ The present study overcame that limitation by providing evidence that BMI had a significantly lesser agreement with DEXA-measures total and trunk fat mass when compared with WHtR.

In a recent systematic review and meta-analysis of 21 cross-sectional studies published in 2023 conducted in the Chinese population on the association of BMI and DEXA-measured fat percentage, only 4 studies included children and adolescents.^41^ The study concluded that the average correlation coefficient between BMI and DEXA-measured fat percentage was 0.60 for children and adolescents.^41^ In contrast, in the present study the cross-sectional correlation between BMI and fat mass ranged between 0.89 and 0.95 in British children and adolescents. This significant difference in correlation may be related to the anthropometry and body composition of different ethnic groups, measures of fat either as percentage or mass in kg, or different DEXA equipment such as General Electric or Norland scanners.^41^ Nonetheless, several studies have shown that increased BMI from childhood was associated with cardiovascular risks in adulthood, however, emerging studies with DEXA measures reported that those associations were likely due to an increase in lean mass rather than fat mass.^2,9,31,32,42^

BMI likely underestimates obesity, particularly in children and adolescents who are sedentary and who might potentially have a lower lean mass for their age, lower body weight, and therefore lower BMI.^43^ However, a recent longitudinal study in >6000 children followed up from ages 11 to 24 years reported that increased sedentary time from childhood was associated with an increased BMI (standard deviation score, SDS = 0.019), increased total fat mass (SDS = 0.019), and increased lean mass (SDS = 0.033).^11^ On the other hand, increased moderate-to-vigorous physical activity was associated with a reduced BMI (SDS = −0.010), reduced total fat mass (SDS = −0.027), and increased lean mass (SDS = 0.006).^11^ The increase in BMI attributed to sedentariness appears to increase lean mass two-fold more than fat mass.^11^ Thus, children and adolescents who are sedentary accumulate more fat, lean mass, weight, and BMI compared to active children. These findings amplify the call to revisit anthropometric indicators in the pediatric population for better surveillance of overweight and obesity due to the poor discriminating ability of BMI.^11,23^

### Waist-to-height ratio as a specific inexpensive tool for predicting fat mass adiposity

The relative stability of WHtR irrespective of age and sex is known and confirmed in the present study.^17,44^ A cross-sectional study of 5355 children aged 8 to 19 years from the US National Health and Nutrition Surveys reported that WHtR performed better than BMI in detecting high body fat percentage.^21^ A cross-sectional survey of 14,042 Chinese students aged 6–17 years concluded from receiver operating curve analyses that WHtR screened children for obesity better than BMI.^45^ The present study validates WHtR cutpoint of 0.50 in males and 0.52 in females as longitudinally optimal to truly identify 4–5 of 10 participants who were overweight or obese (sensitivity) and 9 out of 10 participants who were not overweight/obese (specificity). These WHtR cutpoints in the present longitudinal study represented the 75th percentile of total fat mass in both males and females during growth from childhood through young adulthood. A slightly higher WHtR cutpoint of 0.53 in males and 0.54 in females may potentially identify 8 of 10 participants who were overweight or obese at 95th percentile of total fat mass and nine out of ten participants who were not overweight/obese. Among 9–11-year-old children from Japan, WHtR 85th percentile suggested cutpoint for detecting high DEXA-measured fat mass was 0.47 in males (*n* = 226) and 0.46 in females (*n* = 196). These values were slightly lower than the WHtR cut-points in the present study likely due to participants’ age, sample size (more than 10-fold smaller), and distinctive ethnic-characterized anthropometry and body composition.^46^ Cross-sectional evidence suggests that WHtR may not accurately predict the risk of insulin resistance in Brazilian children and elevated blood pressure in German children.^47,48^ This may suggest that WHtR may be specific for predicting fat mass adiposity. A recent cross-sectional analysis of 24,605 children and adolescents aged 6–18 years from Brazil, China, Greece, Iran, Italy, Korea, South Africa, Spain, the UK, and the USA found that WHtR cutpoint of 0.50 in European and the US youths and 0.46 in Asian, African, and South American youths optimally predicted ≥2 cardiometabolic risk factors.^25^ The combination of WHtR in predicting DEXA-measured total and trunk fat mass with a recent cross-country-validated equation for predicting deuterium dilution fat-free mass could be useful in the global surveillance of fat mass overweight and obesity in the pediatric population.^49^ With WHtR as a better surrogate estimate than BMI in measuring adiposity, future research investigating the mechanism regarding how WHtR correlates highly with fat mass and not with lean mass is warranted. Moreover, the mechanism that explains the relatively unchanged WHtR in a normal growing child until young adulthood in spite of varying BMI necessitates further studies.

### DEXA-measured total fat mass, trunk fat mass, and lean mass percentile cutpoints

The importance of body composition normative reference data from gold-standard measures was highlighted a decade ago among 533 British participants aged 5–20 years.^16^ However, the study did not assess waist circumference or WHtR.^16^ The present study with 14-fold more participants provides comprehensive gold standard reference measures of body composition that could be physiologically and clinically useful in understanding growth, health, and disease.^15^ Importantly, the previous reference values were significantly different, for instance, the total fat mass 50th percentile in males and females at age 10 years was 6.10 kg and 8.89 kg, respectively. In the present study, the total fat mass 50th percentile is 8.36 kg in males and 11.28 kg in females at an average of 9.9 years.^16^ The difference may be attributed to the combination of several measures of body composition such as DEXA, magnetic resonance imaging, and bio-impedance in a calculated 4-component model.^16^

### Strength and limitation

The present study participants were population-representative healthy volunteers participating in an ongoing well-phenotyped prospective birth cohort study (ALSPAC) with repeated and precise gold standard measures of body composition throughout the follow-up period. This extensive data is important in providing normative reference body composition values during growth. Absolute measures of body composition assessed with DEXA presented in this study are more accurate than estimations of fat percentages or equations-derived measures.^49^ The absolute agreement analysis better informs the predictive ability of BMI in identifying fat mass rather than correlation or regression analyses.^13^ A limitation includes the homogeneity of the study population’s race (Caucasian) warranting confirmation in longitudinal studies among other racial backgrounds.^13,17,41^

## Conclusion

WHtR is a better adiposity surrogate measure than BMI in predicting total and trunk fat mass and discriminating lean mass in the pediatric population, and it is unaffected by age and sex. These findings amplify the call to shift away from proxy weight-for-height indices such as BMI, which misidentify and misdiagnose pediatric population with overweight or obesity, and towards a more accurate assessment of fat mass obesity. In males, WHtR cutpoint of 0.50–0.53 could detect 5–8 individuals out of 10 who are truly obese (sensitivity) and 9 out of 10 individuals who are truly not obese (specificity). In females, WHtR cutpoint of 0.52–0.54 could detect 4–7 individuals out of 10 who are truly obese (sensitivity) and 9 out of 10 individuals who are truly not obese (specificity). WHtR may be universally adopted as non-invasive and inexpensive fat mass overweight and obesity surveillance, monitoring, and prevention initiatives in routine pediatric healthcare practice, particularly, in low-resource settings where more complex fat mass measures are not readily available.

## Supplementary information

Supplementary information

## Data Availability

The informed consent obtained from ALSPAC participants does not allow the data to be made freely available through any third-party maintained public repository. However, data used for this submission can be made available on request to the ALSPAC Executive. The ALSPAC data management plan describes in detail the policy regarding data sharing, which is through a system of managed open access. Full instructions for applying for data access can be found here: http://www.bristol.ac.uk/alspac/researchers/access/. The ALSPAC study website contains details of all the data that are available (http://www.bristol.ac.uk/alspac/researchers/our-data/).
