# Supplementary material for: Waist-circumference-to-height-ratio had better longitudinal agreement with DEXA-measured fat mass than BMI in 7237 children
Source: Pediatr Res. 2024 Mar 5;96(5):1369–80. doi: 10.1038/s41390-024-03112-8 (PMC11522001; doi:10.1038/s41390-024-03112-8)
Supplement: Supplementary file 1 — Supplementary information [file 41390_2024_3112_MOESM1_ESM.pdf]

Waist-circumference-to-height-ratio had better longitudinal agreement with DEXA-measured fat mass than BMI in 7237 children

Andrew O. Agbaje MD, MPH[1,2]

[1]*Institute of Public Health and Clinical Nutrition, School of Medicine, University of Eastern Finland, Kuopio, Finland*

[2]*Children's Health and Exercise Research Centre, Department of Public Health and Sports Sciences, Faculty of Health and Life Sciences, University of Exeter, Exeter, UK.*

Address correspondence to:
Andrew O. Agbaje, MD, MPH, Cert. Clinical Research (Harvard)
Institute of Public Health and Clinical Nutrition, School of Medicine, Faculty of Health Sciences, University of Eastern Finland, Kuopio Campus.
Address: Yliopistonranta 1, P.O. Box 1627, 70211 Kuopio, Finland
E-mail: andrew.agbaje@uef.fi

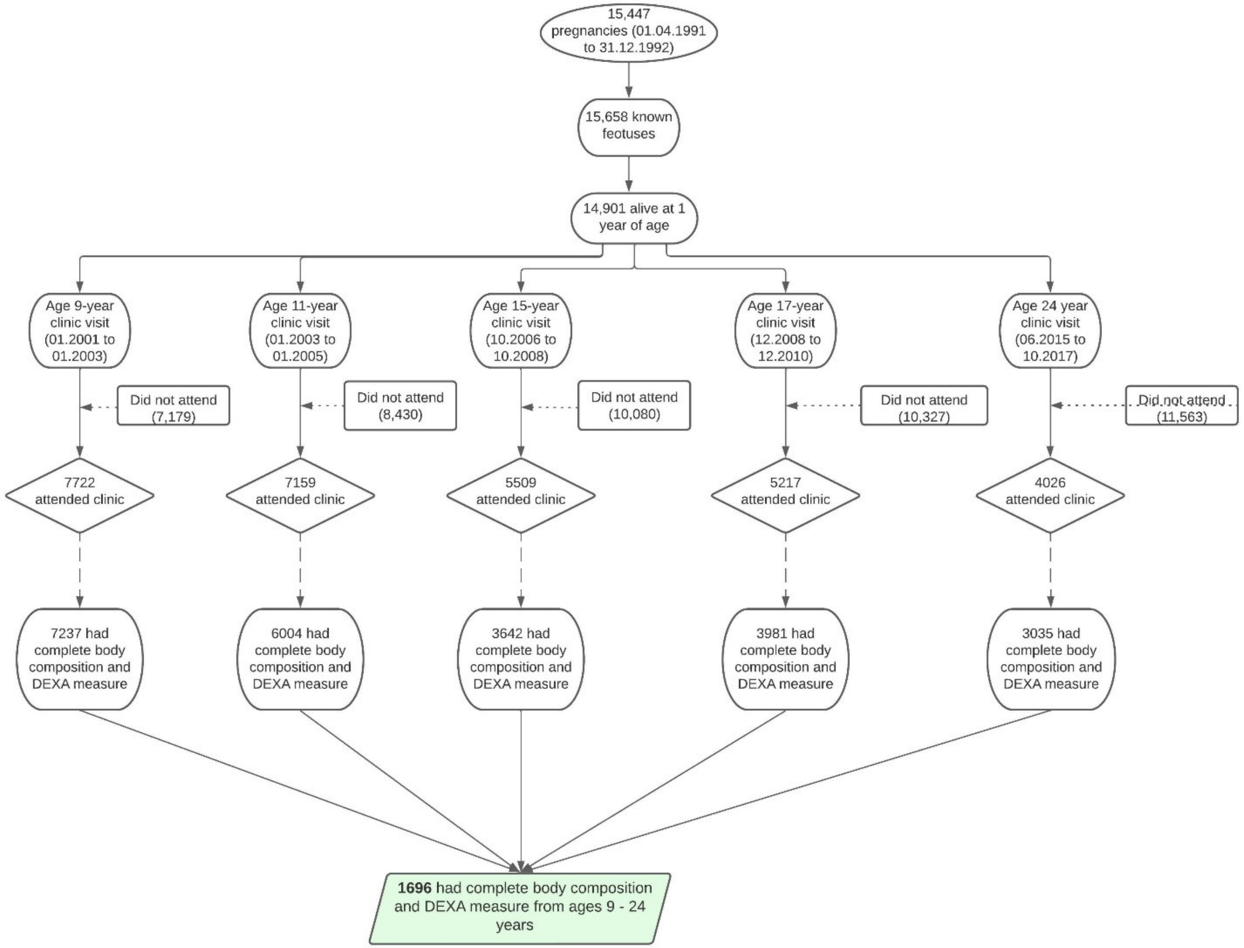

Supplemental Figure 1 Descriptive characteristics of cohort participants.
